# Predictors of Fatigue Among Patients on Hemodialysis: An Observational Study

**DOI:** 10.7759/cureus.65953

**Published:** 2024-08-01

**Authors:** Alessia Faioli, Giorgio Bergesio, Carmen Samà, Benedetta Gallo

**Affiliations:** 1 Department of Long-Term Care, Hospital Cardinal Massaia, Asti, ITA; 2 Department of Sciences of Public Health and Pediatrics, University of Turin, Asti, ITA; 3 Department of Orthopedics, Hospital Cardinal Massaia, Asti, ITA; 4 Department of Long-Term Care, Rehabilitation and Physical Therapy Center of the Turati Foundation, Pistoia, ITA

**Keywords:** post-dialysis fatigue, chronic kidney disease, nurses, hemodialysis, fatigue

## Abstract

Background

Hemodialysis is a chronic replacement therapy recommended during end-stage renal disease (ESRD) in chronic kidney disease (CKD). Post-dialysis fatigue (PDF) is one of the symptoms patients experience after the treatment. This multifactorial condition is subjective and characterized by a lack of physical and emotional energy. It can be associated with other symptoms such as nausea, headache, muscle cramps, and hypotension. Currently, researchers have not been able to find defined etiologies and pathogenesis. This study aims to analyze the influence of PDF predictors within a sample of patients undergoing hemodialysis.

Methodology

A multicenter, observational study was conducted on a convenience sample of 250 CKD patients with ESRD on hemodialysis between February and July 2023. PDF was assessed using the Piper Fatigue Scale (PFS), administered in both paper and electronic formats. The data analysis was done using descriptive and inferential statistics and correlation tests.

Results

The analysis revealed that sleep disorders perceived (p = 0.0001; τ = 0.23), the presence of comorbidity (p = 0.003; τ = 0.18), the number of weekly sessions (p = 0.012; τ = 0.15), and the period of hemodialysis (p = 0.0069; τ = 0.15) had a positive correlation with the PFS score. The study highlighted that hemoglobin level (p = 0.017; τ = - 0.14) and sex (p = 0.012; τ = 0.15) affected the PDF perception. Patients who engaged in physical activities or used distraction techniques (p = 1.83e-05; τ = 0.26) to alleviate PDF reported lower average scores on the PFS (2.27 points) compared to those who did not engage in any such activities.

Conclusions

Hemodialysis appears to be a source of stress for most patients, as they showed a moderate-to-severe score on the PFS scale. PDF is a multifactorial problem that must be recognized and treated properly by nurses through pharmacological therapies, as well as by educating and providing alternative strategies, that can contribute to alleviating the effects of fatigue.

## Introduction

Hemodialysis is the most used treatment for patients affected by chronic kidney disease (CKD). Worldwide, approximately four million people receive renal replacement therapy, with 69% undergoing hemodialytic treatment [1]. Dialysis is a therapy recommended in end-stage renal disease, when the organ becomes significantly compromised, causing catabolite accumulation and water retention, further worsening the patient’s clinical picture and potentially resulting in mortality. The dialyzer filters the patient’s blood using a non-natural membrane retaining catabolites, ions, and water before reintroducing it into circulation through an arteriovenous fistula or a central venous catheter. Normally, each session lasts from three to four hours and is performed three times a week. Hemodialysis has significant implications for the quality of life due to collateral effects (headaches, itching, fatigue, muscle cramps, and hypotension) and to the patient’s reliance on the dialyzer [1]. Fatigue is a symptom that currently lacks a defined etiology and pathogenesis. Post-dialysis fatigue (PDF) is a subjective condition, described by patients as an unpleasant, destructive, and debilitating feeling that results in a lack of physical and mental energy [2]. The literature suggests that the PDF is part of a broader complex of symptoms, including nausea, headache, muscle cramps, and hypotension. These conditions are associated with several factors, such as the displacement of body fluids during a hemodialysis session and the chosen ultrafiltration rate in the treatment [3].

Thus, it becomes crucial to recognize, evaluate, and provide alternative solutions to pharmacological treatments for mitigating PDF. Physical activity has been shown to alleviate this discomfort, but guidelines for healthcare professionals are yet to be established [3].

This study aims to analyze the influence of PDF predictors within a sample of patients undergoing hemodialysis. The results will contribute to a more accurate estimation of the incidence of this phenomenon and may lead to the proposal of potential interventions to alleviate these symptoms.

## Materials and methods

From February to July 2023, a multicenter observational study was conducted on a convenience sample comprising 250 patients suffering from CKD undergoing hemodialysis treatment. Patients treated with peritoneal dialysis were excluded from the analysis. The recruitment of suitable participants for the sample required the collaboration of the Associazione dei Pazienti Emodializzati e Trapiantati (ANED), operating across the entire Italian territory. To assess predictors of PDF in hemodialysis patients, the Piper Fatigue Scale (PFS), validated in Italian, was employed [4]. This tool consists of 22 items measured on a scale ranging from 0 to 10, evaluating four subjective dimensions related to PDF. Questions 2 to 7 evaluate the severity of experienced fatigue, questions 8 to 12 probe the meaning of fatigue for patients, questions 13 to 17 assess fatigue perception, and questions 18 to 23 evaluate their mood. These four dimensions are used to calculate four subscales and the overall PDF score. The final PFS score is calculated by summing the scores of all individual items and dividing by 22. A total score of 0 indicates the absence of fatigue, 1 to 3 indicates mild fatigue, 4 to 6 indicates moderate fatigue, and 7 to 10 indicates severe fatigue. The scale also includes additional items (1, and questions 24 to 27) that evaluate the onset time of PDF, its duration, the perceived probable cause, the presence of other symptoms, and alternative activities employed to alleviate the symptom. These items are not used to calculate the score. The PFS scale was administered both in paper and electronic formats using Google Forms within ANED groups.

Data processing was conducted using R Studio and Microsoft Office Excel (Microsoft Corp., Redmond, WA, USA), employing both descriptive and inferential statistical methods. The initial step involved evaluating the correlation between the independent variables and the PFS score. A correlation matrix was generated using the Kendall concordance coefficient, with a significance level set at p-values of 0.05. The normality of distribution was assessed using the Shapiro-Wilk test. Differences between the PFS score and the independent variables were analyzed using analysis of variance. To further evaluate these differences, the Tukey test and the Welch two-sample t-test were employed to calculate the average scores for each variable subgroup. All participants voluntarily took part in the study and provided their consent. Data collection and processing were conducted anonymously and solely for statistical purposes.

## Results

The examined sample consisted of 250 patients, with an average age of 61 years (SD = ±13.3; range = 47.8-74.5), of whom 60% were males. Among the female population, 28% were fertile while the remaining were in menopause (72%). Analyzing educational background, 45% had obtained a high school diploma, 27% had completed lower secondary school, 14% had attended elementary school, and the remaining 14% held a bachelor’s degree. Within the sample, 68% were unemployed. Regarding marital status, 63% were married, 23% were single/unmarried, and 14% were widowed. Approximately 55% reported having no sleep disturbances, and 61% had comorbidities. Of the participants, 35% had been undergoing dialysis for over six years, 48% for two to five years, and the remaining 17% for less than one year. Overall, 79% of the patients underwent three dialysis sessions per week (Table 1).

**Table 1:** Sociodemographic characteristics of the sample. n = 250 patients; x  = mean; SD= standard deviation

Variable	-	-
	x (±SD)	Range
Age (years)	61 (±13.3)	74.5–47.8
	n (%)	-
20–40	10% (26)	-
41–60	36% (90)	-
61–80	54% (134)	-
Sex		
Males	60% (151)	-
Females	40% (99)	-
Education		
Elementary school	14% (35)	-
Lower secondary school diploma	27% (68)	-
High school diploma	45% (112)	-
Degree	14% (35)	-
Employment		
Yes	32% (81)	-
No	68% (169)	-
Marital status		
Married	63% (158)	-
Single	23% (57)	-
Widow/Widower	14% (35)	-
Fertility		
Period	28% (28)	-
Menopause	72% (71)	-

Laboratory results indicated that 61% of the sample had an albumin level of 5.5 mg/dL or higher, and 68% had hemoglobin levels greater than 10 mg/dL. The study sample (n = 250) had an average PFS score of 2.12 points (SD = ±0.58; range = 1.5-2.7). Among the participants, 65% scored between 4 and 6, 24% scored between 7 and 10, and 12% scored between 1 and 3. Additionally, 52% of the participants reported using non-pharmacological methods to reduce post-hemodialysis fatigue (Table 2).

**Table 2:** Clinical features of the study sample (n = 250). x  = mean; SD = standard deviation; PFS = Piper Fatigue Scale

Variable	-	-	-
Sleep disorders	n (%)	x (±SD)	Range
Yes	45% (112)	-	-
No	55% (138)	-	-
Comorbidity			
Yes	61% (153)	-	-
No	39% (97)	-	-
Number of weekly sessions			
≤2/week	21% (53)	-	-
≥3/week	79% (197)	-	-
Number of years on dialysis			
≤1 year	17% (42)	-	-
2–5 years	48% (121)	-	-
≥6 years	35% (87)	-	-
Albumin			
≤3.5 mg/dL	39% (97)	-	-
≥5.5 mg/dL	61% (153)	-	-
Hemoglobin			
≤9 mg/dL	32% (80)	-	-
≥10 mg/dL	68% (170)	-	-
PFS score	-	2.12 (±0.58)	1.5–2.7
0–3 points	12% (29)	-	-
4–6 points	65% (162)	-	-
7–10 points	24% (59)	-	-
Distractive/Sports activities			
Yes	52% (121)	-	-
No	48% (129)	-	-

**Figure 1:**
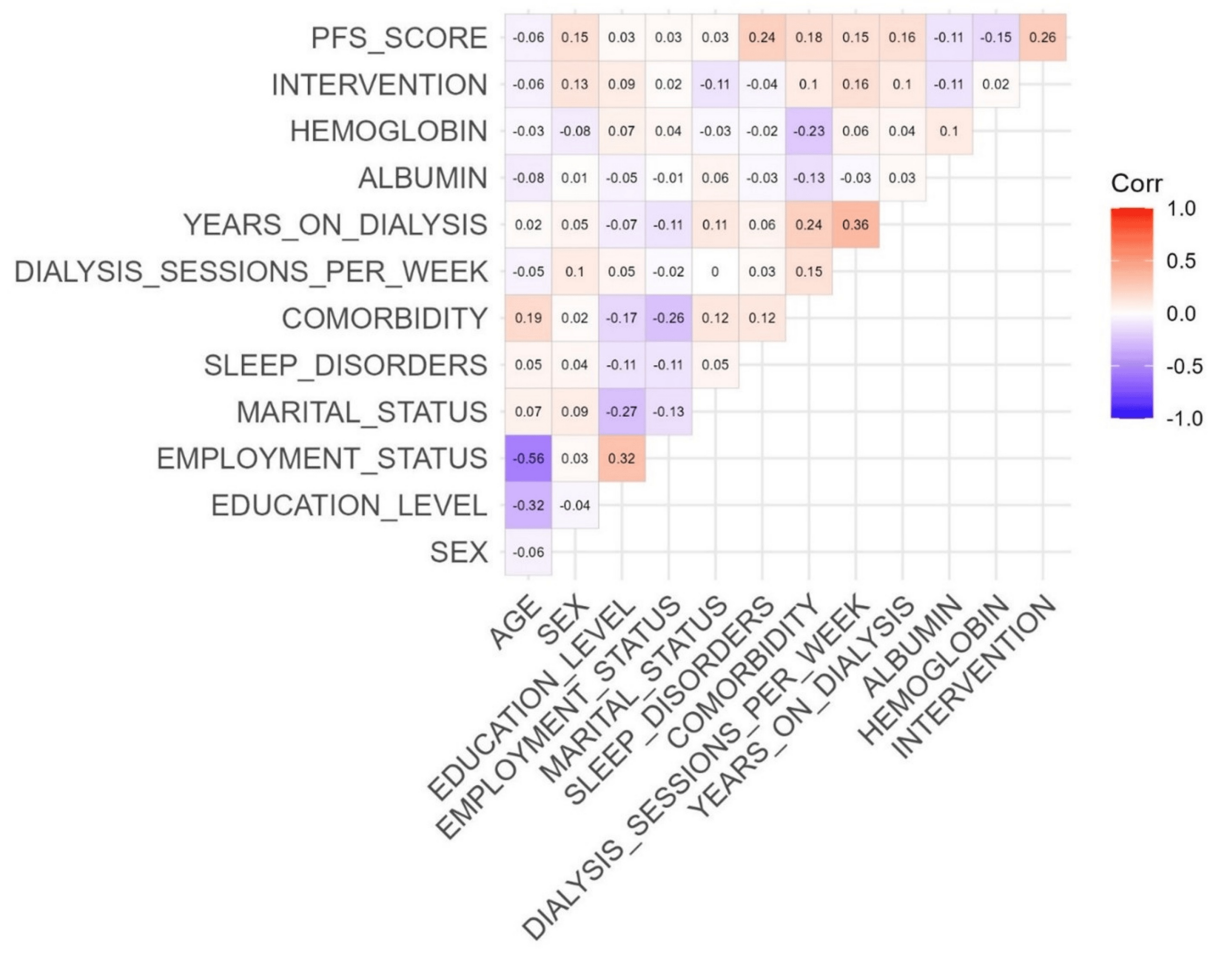
Correlation matrix of all variables. A correlation matrix was generated to evaluate the correlation between the independent variables and the PFS score. Statistical significance was achieved at a p-value <0.05. PFS = Piper Fatigue Scale

Data analysis revealed no correlation between the PFS scale score and the age of the sample (p = 0.27; τ = -0.06), unlike gender, where the association was positive (p = 0.012; τ = 0.15). Other variables, such as educational background (p = 0.60; τ = 0.02), occupation (p = 0.60; τ = 0.03), and marital status (p = 0.56; τ = 0.03), showed no significant correlation with the PFS score. As the PFS score increased, there was a proportional increase (0.23%) in sleeping disorders (p = 0.0001; τ = 0.23), and patients with comorbidities tended to show a higher fatigue level (p = 0.003; τ = 0.18). In the female population, fertility did not appear to impact the level of fatigue (p = 0.52; τ = 0.06). The number of weekly dialysis sessions (p = 0.012; τ = 0.15) and the period of the dialysis (p = 0.0069; τ = 0.15) seemed to increase the PFS score. The albuminemia value did not affect the fatigue perception (p = 0.085; τ = - 0.10). The only laboratory variable with which a positive correlation was observed was hemoglobin. As hemoglobin levels decreased, the perceived PFS score increased (p = 0.017; τ = - 0.14). Patients who engaged in distracting or physical activities to reduce PDF exhibited a higher PFS score (p = 1.83e-05; τ = 0.26) (Figure 1).

In the intravariable analysis, no significant differences were found within subgroups concerning age (p = 0.33), educational background (p = 0.68), occupation (p = 0.59), marital status (p = 0.47), female fertility (p = 0.56), and albumin level (p = 0.10). The statistically significant variables were gender (p = 0.013), perceived sleeping disorders (p = 0.0001), comorbidities (p = 0.0022), number of weekly dialysis sessions (p = 0.012), treatment duration (p = 0.005), hemoglobin level (p = 0.015), and patient interventions to reduce PDF (p = 1.63e-05).

Subgroup analysis showed that the female population tended to have higher average PFS scores compared to males. It was 2.04 points (SD = ±0.57; range = 1.4-2.6) for males and 2.23 points for females (SD = ±0.58; range = 1.6-2.8). Patients who did not report sleeping disorders were prone to have an average PFS score of 1.99 points (SD = ±0.56; range = 1.4-2.5), whereas those who presented such a symptom had a score of 2.27 points (SD = ±0.57; range = 1.7-2.8; diff = 0.28). Comorbidities, the number of weekly sessions, and the years of dialysis led to a higher average on the fatigue assessment scale. Specifically, people with chronic conditions had an average PFS of 2.21 points (SD = ±0.61; range = 1.6-2.8; diff = 0.22); patients undergoing three or more weekly sessions had an average of 2.16 points (SD = ±0.58; range = 1.5-2.7; diff = 0.22), and with increasing years of dialysis, the PFS score also increased. Patients who attended hemodialysis for more than six years had an average PFS of 2.22 points (SD = ±0.59; range = 1.6-2.8), while those who were under treatment for less than a year presented an average of 1.90 points (SD = ±0.57; range = 1.3-2.4; diff = 0.31; p = 0.011). Values of hemoglobin less or equal to 9 mg/dL increased the fatigue perception in patients with an average scale score of 2.25 points (SD = ±0.54; range = 1.7-2.7; diff = 0.19).

Patients involved in distracting or physical activities to reduce PDF showed an average PFS score of 2.27 points (SD = ±0.58; range = 1.6-2.8) compared to those who did not employ any strategy (SD = ±0.53; range = 1.4-2.4; diff = 0.31). The results of the intravariable analysis are summarized in Table 3.

**Table 3:** Summary of intravariable analysis. The independent variables were analyzed using the analysis of variance test, Tukey test, and the Welch two-sample t-test. SD = standard deviation; PFS = Piper Fatigue Scale

Variable	P-value	Intravariable difference	Mean PFS score (SD)	Range
Age	0.33	-	-	-
Sex	0.013	0.18	-	-
Males	-	-	2.04 (± 0.57)	1.4–2.6
Females	-	-	2.23 (±0.58)	1.6–2.8
Education	0.68	-	-	-
Marital status	0.47	-	-	-
Employment	0.59	-	-	-
Fertility	0.56	-	-	-
Sleep disorders	0.0001	0.28	-	-
Yes	-	-	2.27 (±0.57)	1.7–2.8
No	-	-	1.99 (±0.56)	1.4–2.5
Comorbidity	0.002	0.22	-	-
Yes	-	-	2.21 (±0.61)	1.6–2.8
No	-	-	1.98 (±0.54)	1.4–2.5
Number of weekly sessions	0.012	0.22	-	-
≤2/week	-	-	1.94 (±0.53)	1.4–2.4
≥3/week	-	-	2.16 (±0.58)	1.5–2.7
Number of years on dialysis	0.0057	-	-	-
≤1 year	0.086	-	1.9 (±0.57)	1.3–2.4
2–5 years	0.011	-	2.12 (±0.55)	1.5–2.6
≥6 years	0.47	-	2.22 (±0.59)	1.6–2.8
Albumin	0.10	-	-	-
Hemoglobin	0.015	0.19	-	-
≤9 mg/dL	-	-	2.25 (±0.54)	1.7–2.7
≥10 mg/dL	-	-	2.06 (±0.59)	1.4–2.6
Distractive/Sports activities	1.63e-05	0.13	-	-
Yes	-	-	2.27 (±0.58)	1.6–2.8
No	-	-	1.96 (±0.53)	1.4–2.4

## Discussion

This study aimed to correlate the impact of certain variables with the fatigue level within a sample of hemodialysis patients (n = 250). First, enrolled patients were asked to identify, through a series of open-ended questions, the peak time of fatigue incidence, the perceived cause, personal intervention strategies, and the presence of additional symptoms beyond those covered by the PFS. Most patients experienced fatigue minutes or hours after the treatment, which typically decreased by the following day, eventually disappearing completely. For this reason, patients preferred afternoon sessions to allow for full recovery during nighttime rest. Half of the sample referred to using coping strategies to alleviate fatigue, with the most common being post-dialysis rest, while a minority engaged in physical activities such as walking for about 30 minutes, various forms of exercise, socializing, or engaging in recreational activities.

According to Horigan and colleagues, nurses play a crucial role in identifying PDF using assessment tools that analyze both the severity of symptoms and their impact on the patient’s quality of life. It is important to monitor PDF recovery times, evaluate the collateral effects of pharmacologic therapy, educate the patient on proper diet, and perform functional physical exercises to reduce strain [5,6].

The diet should be chosen according to dry weight, as proper weight leads to better blood pressure management, a well-functioning cardiovascular system, and improved tolerance during sessions. It is necessary to control the fluid and sodium intake, increase protein intake, and reduce foods high in potassium and phosphorus to prevent cardiovascular problems. However, collaboration with a dietitian to plan balanced and healthy meals tailored to the patient’s clinical condition is necessary [6,7].

In the study sample, patients with moderate-to-severe PFS scores engaged in distracting or physical activities, in contrast to those who tolerated fatigue more easily and did not engage in any treatment. A literature review revealed that patients who engaged in any form of physical activity at least twice a day on non-dialysis days experienced shorter recovery times from PDF [5]. Physical activity, regardless of its simplicity, appeared to be one of the most effective methods for combating and reducing the sense of fatigue in patients. Several randomized studies concluded that low-to-moderate-intensity physical activity performed before or during a session significantly decreased general fatigue. However, the research did not provide sufficient data regarding improving PDF recovery times [5].

Furthermore, participating in any sports activity led to improvements in insomnia and reduced pain perception, fatigue, and depression. To improve sleep quality, patients need to eliminate caffeine, nicotine, and alcohol intake and stay awake during dialysis [6]. In another study, two groups of people were analyzed: the first consisted of individuals with a daily program that included two 10-minute walking sessions on non-dialysis days for six months, while the second was a control group with no planned activity. The results indicated that the exercise group reported significantly reduced fatigue levels and shorter recovery times, whereas these factors remained unchanged for the control group. These results do not confirm the effectiveness of constant physical activity, but they highlight improvements in the perception of fatigue without any adverse effects [8]. Subsequently, results from the PFS were analyzed and correlated with the sample variables. Data analysis revealed that the majority of participants perceived the symptomatology associated with PDF as a moderate disorder. This finding is consistent with the literature and is likely related to the adaptive capacity that patients with chronic conditions develop over time. PDF is indeed a widespread condition among hemodialysis patients who experience physical (asthenia, loss of energy), emotional (social isolation, depression), and cognitive (concentration and memory difficulties) symptoms [2,9]. In more severe cases, it has a negative impact on daily life, leading to social isolation or loss of autonomy [7,10,11].

In this study, the females seemed to be more predisposed to fatigue than males, confirming reports from other studies. This could be because women, despite having debilitating chronic conditions, continue to manage household and family responsibilities, subjecting themselves to greater stressors [2,9,11,12]. Although the literature linking fertility and PDF is limited, some studies argue that menopause may predispose women to the symptoms. The data analyzed in this study had no significant value, as the case mix was different compared to those of other studies that enrolled only women [9]. However, menopause as a predisposing factor cannot be excluded as the subjects are undoubtedly older and the decreased production of estrogen may lead to increased fatigue. Another factor that can affect the analyzed phenomenon was the relationship between sleeping disorders and fatigue, which appeared dual, as a disruption of the normal rest cycle can predispose to chronic fatigue, and vice versa, with this symptom blocking the proper sleep-wake cycle. In the analyzed sample, it was possible to confirm this correlation resulting in repeated nighttime awakenings, difficulty falling asleep, and difficulty resuming sleep. In both cases, patients were subjected to rest depletion stress. It is, therefore, important that healthcare professionals systematically evaluate the quality of patients’ sleep and provide coping strategies to prevent and treat this problem [2,9,13,14]. It can be asserted that, as is evident that sleeping disorders significantly impact PDF, the presence of comorbidities also negatively affects the quality of life of hemodialysis patients [7,15,16]. In fact, polytherapy predisposes to side effects or pharmacological interactions, especially in elderly individuals with multiple chronic conditions. Being polypathological reflects both physically and emotionally, increasing the stress condition. In the analyzed sample, there was a positive correlation between comorbidities and PDF, confirming other previous studies [2,9,15].

Although the literature is not unanimous in associating hemoglobin levels with PDF symptoms, it was plausible to expect that an anemic condition would lead to general hypoxemia, resulting in decreased exercise tolerance. In the conducted study, the association between the two symptoms indeed showed a positive correlation, and, most likely, authors who previously studied this relationship considered severe symptoms or enrolled patients with normal hemoglobin levels [11]. Another laboratory parameter considered to assess its incidence was albumin. Studies have reported that patients with hypoalbuminemia had higher levels of chronic fatigue [2,9]. The results regarding the correlation between albuminemia and fatigue mentioned above did not overlap in this analysis, even though it is conceivable that a decrease in albumin could disrupt the balance of intravascular and extravascular fluids, leading to hypovolemia and edema. Preventing malnutrition and ensuring proper protein and vitamin intake represent a way to prevent hypoalbuminemia. Furthermore, an increase in weight (brought to the session) resulted in a greater amount of fluids being removed, and due to the need for a higher dialyzer filtration rate, hypovolemia could occur, leading to hypotension. This observation was confirmed by direct feedback from patients who arrived with lower weight on the treatment day, experiencing lower levels of PDF. Therefore, it can be assumed that weight may also influence symptom perception. This result could be influenced by different normal levels chosen by the panel of researchers or the nutritional status of the analyzed samples. Similarly, in this study, age was not found to be a significant predictor of PDF perception. The data obtained differ from those reported by other studies, presumably because the average age of the analyzed samples was different, and the sample size was smaller [9]. However, it is not to be excluded that older patients may perceive the symptom with greater intensity, as the physiological aging process makes their bodies more fragile and susceptible to multifactorial changes. Additionally, the elderly often suffer from malnutrition and therefore have a higher likelihood of experiencing fatigue [7,14,15,17,18]. In the literature, it has been noted that marital status has a positive correlation with the perception of physical and mental fatigue. Married individuals tend to experience more fatigue, and females appear to be at greater risk compared to singles and males. It could be inferred that assistance from a partner at home can make life easier, even if only by contributing to household chores and task division, thus reducing stress and resulting in lower perceived fatigue [14]. Discordant results are found in studies that have considered employment status [12]. In the study by Joshwa and colleagues, unemployment seemed to lead to negative feelings in patients, worsening the perception of PDF [14]. Similarly, the level of education was found to have a positive correlation, but in this case, it improved the perceived PDF. In particular, the severity of perceived PDF was inversely proportional to education level, likely due to a limited or complete lack of knowledge [9,10,14]. For both of the variables mentioned earlier, this investigation did not reveal a correlation with the PFS score.

In this study, patients undergoing peritoneal dialysis were excluded; therefore, it might be interesting to investigate PDF in this group of individuals. Additionally, patients with cognitive impairments were also excluded. The observational study conducted has other limitations, such as the small sample size. Moreover, for the variable of years on dialysis, as close-range years were chosen, a different division could yield different results.

## Conclusions

Patients undergoing hemodialysis are compelled to make a radical lifestyle change as they are obliged to experience a chronic and definitive replacement treatment. Such therapy appears to be a source of stress for most patients, as they showed a moderate-to-severe score on the PFS. PDF is a multifactorial problem that must be recognized and treated properly by nurses, not only through the usage of pharmacological therapies but also by educating and providing alternative strategies that can contribute to alleviating the effects of fatigue. Further studies with larger sample sizes are needed to confirm the findings of this study.
